# Antibody Cross-Reactivity in Antivenom Research

**DOI:** 10.3390/toxins10100393

**Published:** 2018-09-27

**Authors:** Line Ledsgaard, Timothy P. Jenkins, Kristian Davidsen, Kamille Elvstrøm Krause, Andrea Martos-Esteban, Mikael Engmark, Mikael Rørdam Andersen, Ole Lund, Andreas Hougaard Laustsen

**Affiliations:** 1 Department of Biotechnology and Biomedicine, Technical University of Denmark, DK-2800 Kongens Lyngby, Denmark; lilej@dtu.dk (L.L.); andrea.martos.esteban@gmail.com (A.M.-E.); mikaelengmark@gmail.com (M.E.); mr@bio.dtu.dk (M.R.A.); 2 Department of Veterinary Medicine, University of Cambridge, Cambridge CB3 0ES, UK; tpj24@cam.ac.uk; 3 Computational Biology Program, Fred Hutchinson Cancer Research Center, Seattle, WA 98109, USA; kdavidse@fredhutch.org; 4 Department of Bio and Health Informatics, Technical University of Denmark, DK-2800 Kongens Lyngby, Denmark; kamille1212@hotmail.com (K.E.K.); lund@bioinformatics.dtu.dk (O.L.)

**Keywords:** antivenom, cross-reactivity, cross-neutralization, high-density peptide microarray technology, antivenomics, snakebite envenoming, venom, toxins

## Abstract

Antivenom cross-reactivity has been investigated for decades to determine which antivenoms can be used to treat snakebite envenomings from different snake species. Traditionally, the methods used for analyzing cross-reactivity have been immunodiffusion, immunoblotting, enzyme-linked immunosorbent assay (ELISA), enzymatic assays, and in vivo neutralization studies. In recent years, new methods for determination of cross-reactivity have emerged, including surface plasmon resonance, antivenomics, and high-density peptide microarray technology. Antivenomics involves a top-down assessment of the toxin-binding capacities of antivenoms, whereas high-density peptide microarray technology may be harnessed to provide in-depth knowledge on which toxin epitopes are recognized by antivenoms. This review provides an overview of both the classical and new methods used to investigate antivenom cross-reactivity, the advantages and disadvantages of each method, and examples of studies using the methods. A special focus is given to antivenomics and high-density peptide microarray technology as these high-throughput methods have recently been introduced in this field and may enable more detailed assessments of antivenom cross-reactivity.

## 1. Introduction

Snakebite envenoming was added to the list of the world’s most neglected tropical diseases by the World Health Organization in 2017 [1]. This has renewed the interest in antivenom research and will hopefully lead to new advances within the field. The current treatment of snakebite envenoming is based on polyclonal antibodies obtained from the plasma of animals hyper-immunized with snake venom [2]. In producing such antivenoms, the snake venoms included in the immunization mixtures (homologous venoms) are chosen based on which snakebites the antivenom is intended to treat. However, snake venoms differ greatly, both between different species, as well as between specimens from the same species inhabiting different geographical locations [3,4]. Additionally, the immunogenicity of different groups of snake toxins are highly variable, which can result in antivenoms with variable neutralizing abilities against certain toxins. It is therefore highly desirable for antivenoms to be able to neutralize different toxins in venoms from multiple snake species, a trait called cross-neutralization. As the neutralization capacity of an antivenom is often tested through in vivo animal neutralization assays, these studies are mostly preceded by tests of the ability of the antivenom to cross-react with different snake species, meaning its ability to cross-bind to toxins from several snake species. If the antivenom is found to exhibit cross-reactivity towards venom toxins from snake species the antivenom was not raised against (heterologous venoms), the antivenom is classified as para-specific. The concept of cross-reactivity will be presented and discussed in this review along with a description and discussion of advantages and disadvantages of the different methods used to determine cross-reactivity such as immunodiffusion, immunoblotting, ELISA, enzymatic assays, in vivo neutralization studies, surface plasmon resonance, antivenomics, and high density peptide microarray.

## 2. The Molecular Basis for Cross-Reactivity

**Figure 1:**
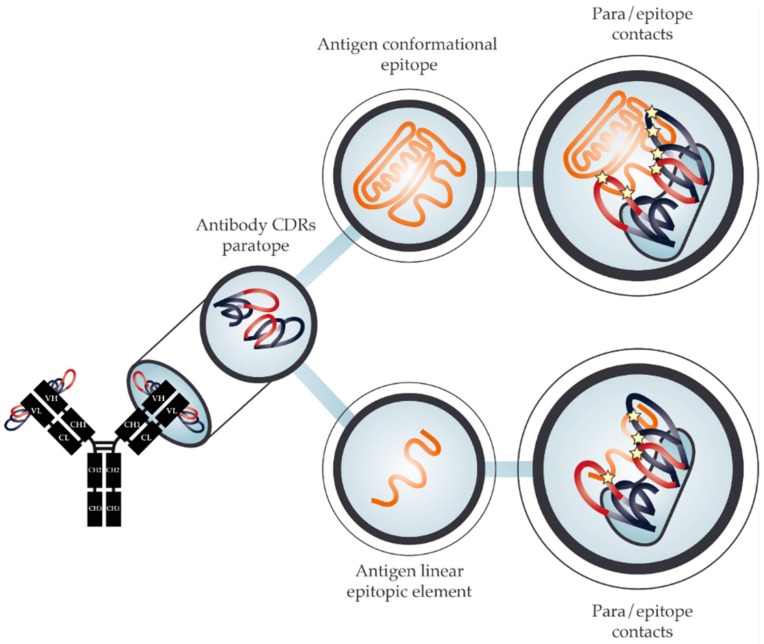
Schematic representation of the paratope-epitope interaction between an antibody binding to an antigen. The antibody paratope is comprised of Complementarity Determining Regions (CDRs). In the upper row, a conformational epitope of the full antigen is bound by the antibody, while in the lower row, a linear epitopic element of said epitope is bound in a similar fashion, but with fewer points of interaction (stars).

In response to an antigen challenge, such as a venom injection, the vertebrate adaptive immune system raises antibodies to neutralize the external threat. Toxin neutralization is achieved through antibody binding and either blocking the antigen from exerting its toxic effect or simply sequestering it in circulation until the toxin is destroyed, i.e., by lysosomal degradation [5,6]. Antibodies bind antigens through the interaction of the paratope on the antibody and the epitope on the antigen. The paratope is typically confined to the complementarity-determining regions (CDRs) on the antibody, whereas the antigen can have many epitopes on different parts of its three-dimensional protein surface. All antigen epitopes are conformational in nature. However, this review operates with two practical distinctions relating to how epitopes are detected: (i) conformational epitopes, which are natively shaped epitopes on a fully folded antigen, and (ii) linear epitopic elements, which are continuous linear stretches of amino acids from the antigen sequence or which may be a mimotope, which mimics (part of) the structure of an epitope as seen in Figure 1. A linear epitopic element may indeed cover the full epitope sequence, but may also cover only a part of the epitope (such as a continuous element of a conformational epitope). Despite its name, a linear epitopic element is still bound to its paratope in a three-dimensional conformation, where the peptide can potentially fold into a structure mimicking a conformational epitope, as shown in Figure 1. The advantage of operating with linear epitopic elements is that they are often substantially simpler to use in experiments compared to their conformational counterparts, as they are easy to synthesize and display on various different surfaces. Epitopes in their natural antigen context have been shown to contain continuous linear elements and for most conformational epitopes a single linear element makes up the majority of the interaction surface [7]. This provides theoretical evidence explaining how linear epitopic elements can often successfully approximate their true conformational epitopes, as observed in several studies [8,9,10]. However, not all epitopes can be approximated using a short linear peptide, as well as binding interactions between paratopes and linear epitopic elements are not necessarily proportional to the affinity between antibody and antigen in their native conformation.

Cross-reactivity is defined as a function persisting across different molecular contexts. Specifically, we refer to two such functions as: antigen binding and antigen neutralization. Antibody-antigen binding is a simple measure, quantifiable through in vitro assays such as enzyme-linked immunosorbent assays (ELISAs) or affinity measurements. On the other hand, antigen neutralization is a measure of an antibody’s ability to protect an organism or a cell from the effector function of the antigen, e.g. neurotoxicity of a three-finger toxin (3FTx). Therefore, neutralization assays are mostly performed in either cell cultures or living organisms, with the exception of toxins where an enzymatic assay is available and correlates with in vivo neutralization [11]. On the level of antigen binding, an antibody may bind to several similar antigens. However, this type of cross-reactivity does not necessarily translate into cross-neutralization, reflecting the complex relationship between antibody affinity and neutralization of toxicity. Although, many studies have reported a positive correlation between affinity and neutralization [12,13,14], several others have demonstrated that this cannot be generalized to all antigens [15,16,17]. When comparing antibodies binding to the same epitope, the correlation between affinity and neutralization seems to be more robust, indicating that the lack of correlation is due to differences in neutralization efficiencies on different epitopes [12,14].

Antibody cross-binding of linear epitopes has previously been observed for snakebite antivenoms. By peptide mutagenesis, it has been shown that cross-binding was driven by a short motif of conserved residues [18], presumably making the most important contacts with the paratope. Linear epitopic elements have often been found to map back to the N/C termini or loop regions of the antigen [8,18]. Similarly, epitopes in their native antigen structures are also reported to be overrepresented within loop regions [19]. However, epitope secondary structure is still likely to be a confounding factor to mapping of linear epitopic elements. It could theoretically be expected that epitopes in unstructured regions are detected with a higher true positive rate than epitopes in structured regions, which on the other hand are expected to be harder to detect and more prone to false negatives. For conformational epitopes, antibody cross-binding is supported by structural similarity of the epitope and key residues contacting the antibody. Protein structure is typically conserved among homologs, even in cases with low sequence similarity [20]. This conservation of structure also extends to the epitopes and provides ample ground for cross-binding, especially in functionally conserved surface areas with conserved residues, such as protein binding sites and accessible catalytic sites.

While antibody cross-reactivity is a useful property in venom neutralization, all snakebite antivenoms currently on the market are plasma-derived polyclonal antibody mixtures with undefined composition. Therefore, in the context of polyclonal antivenoms, cross-reactivity is defined as the ability of the antivenom as a whole to bind multiple different antigens, a desirable quality that is encouraged in antivenom production by utilizing venoms from multiple snake species for immunization [21]. In relation to polyclonal antivenoms, two theoretical mechanisms can explain observed cross-reactivity: (i) Monoclonal antibodies may have cross-binding capabilities towards more than one toxin due to the tolerance of antibodies regarding a certain degree of antigen variation (analogous to broadly neutralizing antibodies of HIV [22] or Influenza [23]). (ii) As antivenoms are polyclonal mixtures essentially comprising large repertoires of monoclonal antibodies that recognize at least one epitope, an antivenom mixture as a whole may derive cross-reactivity from having different monoclonal antibodies that individually bind single toxins present in different venoms [21]. In the latter mechanism, the collection of antibodies will in symphony behave like a cross-reactive antibody, even if no individual monoclonal antibody in the collection is capable of binding more than one specific toxin epitope. Extensive cross-reactivity of antivenom has been observed in several studies [18,24,25,26,27,28,29,30,31], but it remains elusive to which extent each of the two above mechanisms contribute to this observation.

Classically, cross-reactivity of snake toxin-targeting antibodies as well as antivenoms has been difficult to study and has involved laborious isolation procedures for venom toxins and testing of binding through ELISAs or assays based on SPOT synthesized peptide arrays [32]. However, new technologies are expanding the possibilities of studying cross-reactivity in high-throughput. This opens new avenues for guiding the development of improved antivenoms with broader neutralization breadth, but also provides a testing suite for quality and consistency of current commercial antivenoms [18,33]. Expanding the breadth of antivenom neutralization capacity is of special importance, as this can offer full neutralization coverage with fewer antivenoms. Ultimately, mixtures of selected highly cross-reactive monoclonal antibodies may potentially substitute plasma-derived antivenoms, paving the way for a fully recombinant product produced in cell cultures with a consistently high quality [34]. For a more intermediate scope, a possibility also exists to fortify existing plasma-derived antivenoms with single or a few monoclonal antibodies that either broaden the neutralization capacity of the antivenom or improve the efficacy of the antivenom against key toxins.

In the following, we provide an overview of the approaches used to investigate antivenom cross-reactivity towards different toxins and whole venoms. A special focus is granted to antivenomics and high-density peptide microarray technology as these are important methods providing high-throughput solutions to examine and reveal details about antibody cross-reactivity, as well as they are promising tools for efficient characterization of cross-reactive toxin-targeting antibodies.

## 3. Traditional Studies of Cross-Reactivity

Antivenom cross-reactivity can be assessed using several methods, all providing different levels of throughput. Traditionally, the in vitro methods used for determination of cross-reactivity have involved immunoblotting, immunodiffusion, and ELISA. Cross-neutralization is typically examined in vivo by testing whether an antivenom can prevent lethality of different venoms in mice and this method is recognized as the golden standard for determining venom neutralization. In vitro experiments can also be used to estimate neutralization capacity, such as enzymatic assays determining phospholipase A_2_ (PLA_2_) activity, proteolytic activity, and hyaluronidase activity [31]. Cellular in vitro assays assessing e.g., cell viability [35] could also be employed, but are not routinely used to assess cross-neutralization. When evaluating the efficacy of an antivenom against a heterologous venom, in vitro determination of cross-binding is traditionally investigated prior to in vivo studies. In vitro experiments are conducted to limit the number of in vivo tests and to limit the use of animals. Several published studies investigating the cross-reactivity of heterologous antivenoms exist using both in vitro and in vivo experiments.

### 3.1. Immunodiffusion

Immunodiffusion is an older method that has been employed to investigate antivenom cross-reactivity for many decades. The experimental setup is simple and involves diffusion of venom and antivenom in an agarose or agar gel. Precipitation lines will form in the gel if binding occurs between the antivenom antibodies and the venom toxins. An example is provided by Lauridsen et al., who examined cross-binding of the VINS African antivenom and VINS Central Africa antivenom to the venom from *Dendroaspis angusticeps* (green mamba) and *D. polylepis* (black mamba) [36]. Using immunodiffusion, they demonstrated the presence of antibody cross-binding for both of the VINS antivenoms to the venom proteins of *D. angusticeps* and *D. polylepsis*, whereas no cross-binding was detected for an antivenom raised against *M. nigrocinctus* used as a negative control. The results of the immunodiffusion assay did not correctly reflect the neutralizing ability of the antivenoms, as only the VINS African antivenom was shown to prevent lethality in vivo. These findings highlight how immunodiffusion can have merit in the initial determination of cross-binding between a venom or a venom fraction and an antivenom, given the low cost, ease of use, and robustness of this assay. However, the results might not reflect the neutralizing capacity of the assayed antivenoms, and the sensitivity of the assay is not high enough to detect presence of antibodies if their abundance is low. 

### 3.2. Immunoblotting

Immunoblotting is a method used for determining cross-reactivity of antivenoms, while providing more information on the venom toxins recognized by an antivenom compared to immunodiffusion. This method provides information about the molecular weight, isoelectric point, or electric charge of the toxins recognized by the antivenom, dependent on how the toxins are separated. Both one-dimensional (1D) and two-dimensional (2D) immunoblotting have been used for cross-reactivity studies. An example of a study using 1D immunoblotting to assess cross-reactivity is provided by Casewell et al. [29]. This study tested the two monospecific antivenoms, ViperaTAb from MicroPharm Limited and Zagreb from the Institute of Immunology in Zagreb, for their cross-binding abilities towards venoms from European vipers. The immunoblotting results showed that the antivenoms extensively cross-reacted with toxins of different molecular weights in the venoms of *Vipera berus*, *V. aspis*, *V. ammodytes*, and *V. latastei*. When the median effective dose (ED_50_) was determined in vivo, it was discovered that both antivenoms could cross-neutralize all four venoms. However, the ViperaTAb antivenom had an equal or lower ED_50_ compared to the Zagreb antivenom, which was not reflected in the immunoblotting results. Immunoblotting is a more complex assay to conduct than immunodiffusion, but has the advantage of providing more information on the toxins recognized by an antivenom. Similar to immunodiffusion, immunoblotting does not reflect toxin neutralization, but only binding. 

### 3.3. Whole Venom and Venom Fraction ELISA

ELISA is a method of very high sensitivity and can be employed to detect even low levels of antivenom cross-reactivity. ELISA is often used to determine cross-binding of antivenoms to different venoms and involves either whole venom, venom fractions, or isolated toxins. One study by Tan et al. [30] used whole venom ELISA to examine cross-binding of the BioCSL sea snake antivenom (SSAV) to the venom of *Laticauda colubrina*. The results showed similar binding activity between the SSAV antivenom and the *L. colubrina* venom as observed between SSAV and the homologous venoms of *Hydrophis schistosus* and *Notechis scutatus*. Lethality experiments were later conducted in vivo, where the antivenom was shown to prevent lethality induced by *L. colubrina* venom. ELISA experiments utilizing whole venom have the advantage of being easy and quick to perform on many samples, compared to the other in vitro assays described in this section. The higher throughput is obtained by using standardized microtiter plates, which allows the testing of several different venoms against a range of different antivenoms in one experiment. Similar to immunodiffusion, this type of ELISA only explores whether antibodies in the antivenom bind venom components, but does not provide any data on which venom toxins are recognized, unlike immunoblotting. However, the sensitivity of ELISA is significantly higher than immunodiffusion and immunoblotting, and therefore, low levels of cross-reactivity are detectable. 

A more in-depth ELISA experiment can be performed, if more information about which venom toxins an antivenom binds to is desired. In such an ELISA experiment, the antivenom is tested against either venom fractions or individual toxins, instead of whole venom. Apart from immunodiffusion, Lauridsen et al. [36] also used this in-depth ELISA method to examine cross-binding of the VINS African antivenom and VINS Central Africa antivenom towards the venom of *D. angusticeps*. The two antivenoms were tested against 26 *D. angusticeps* venom fractions, providing comparable results. The binding signals for almost all fractions were significantly higher than the signals for the antivenom used as negative control. Though the two antivenoms produced similar results from the ELISA experiment, only the VINS African antivenom could neutralize venom in vivo, as previously mentioned. This was not suprising, as this antivenom was generated using venom from a closely related snake, *D. polylepis,* which was not the case for the VINS Central Africa antivenom.

### 3.4. Enzymatic Assays

Different types of in vitro enzymatic assays can be used to estimate antivenom neutralization of toxins with enzymatic activity. Enzymatic assays are the only in vitro assays in routine use that reveal not only binding, but also inhibition of biological function. This is a significant advantage; however, it can only be applied to studying venoms, where enzymatic toxins contribute significantly to venom toxicity, and it therefore provides limited value for investigation of many elapid venoms, where neurotoxicity drives venom toxicity [6,37]. Enzymatic assays can be used to determine neutralization of PLA_2_ activity, proteolytic activity, and hyaluronidase activity [38,39]. These assays involve addition of a substrate that can be converted to a quantifiably detectable product by the investigated enzymatic toxin and can be conducted in standardized microtiter plates and therefore generate data in high-throughput. Tanaka et al. [31] investigated the neutralization of PLA_2_ activity, proteolytic activity, and hyaluronidase activity and used the information to determine cross-reactivity of the Brazilian coral snake antivenom against seven different heterologous *Micrurus* snake venoms. Here, all three enzymatic activities in venom from *M. ibiboboca* were neutralized by the Brazilian coral snake antivenom, whereas the neutralization of these enzymatic activities was not as high for *M. spixii* venom. However, the in vivo results were poor for antivenom against the two venoms compared to the results obtained from the enzymatic studies. Although enzymatic assays do provide valuable information about neutralization, they are restricted to the investigation of venoms, where enzymatic toxins play a key role in venom toxicity, and the results do not always reflect neutralization of venom in vivo.

### 3.5. In Vivo Experiments

Accurate venom neutralization experiments can only be conducted in vivo, which is why in vitro cross-reactivity experiments should be supported by in vivo data. The golden standard of in vivo neutralization experiments is to determine if an antivenom can prevent venom lethality. Typically, this is done by first determining the median lethal dose (LD_50_) of the venom by injecting mice with different amounts of venom and thereafter testing how much antivenom is needed to neutralize the lethal effects of a set number of LD_50_s. The ED_50_ obtained from these experiments reflects the ability of the antivenom to cross-neutralize venom induced lethality. The ED_50_ is traditionally assessed using preincubation experiments, where venom and antivenom are preincubated before administration. However, this approach does not reflect real life envenomings and rescue experiments. Experiments where antivenom and venom are administered seperatly may generate results more acurately resembeling real life envenomings. Other in vivo experiments can also be employed to assess neutralization of hemorrhagic activity, necrotizing activity, myotoxic activity, and edematogenic activity of venoms [11], as well as in vivo experiments can be modified using different routes of administration, different administration times between venom and antivenom, and individual toxins or venom fractions [40,41]. Although testing of neutralization of these effects to determine the efficacy of an antivenom is recommended, these tests are rarely used in studies of antivenom cross-reactivity. Tan et al. have published several studies where cross-neutralization of different antivenoms to heterologous venoms have been determined using traditional lethality studies [30,40,42]. 

In vivo experiments are the only way to fully determine the (cross-)neutralization potential of antivenoms. However, some of the drawbacks of assessing cross-neutralization through venom lethality experiments are high costs and ethical considerations.

### 3.6. In Vivo versus In Vitro Determination of Cross-Reactivity 

Absence or presence of cross-binding between a venom and an antivenom can be determined by using the in vitro assays; immunoblotting, immunodiffusion, and ELISA experiments. In this way, the methods are useful as they eliminate the need to determine cross-neutralization in vivo, if no cross-binding can be detected in vitro. In cases where cross-reactivity is detected using these methods, it cannot directly be translated into presence of cross-neutralization. Presence of cross-reactivity only reflects that venom toxins are recognized and bound by antibodies in an antivenom. However, it is not unveiled whether the recognized toxins are of medical importance or whether the antibodies recognize epitopes preventing the toxins from exerting toxicity. An in vitro strategy that can be employed to provide more detailed information on which toxins are recognized, and whether or not cross-neutralization potential is present, is the use of ELISA to determine cross-reactivity between an antivenom and individual venom fractions or toxins instead of whole venom. Following this approach, more toxin-centric information is obtained. However, in vivo lethality experiments must eventually be conducted to confirm cross-neutralization.

### 3.7. Surface Plasmon Resonance

The idea behind surface plasmon resonance (SPR) dates back to more than a century ago [43]. However, the technique has only gained wide popularity within the study of antibodies within the last few decades [44]. SPR-based biosensors combine multiple benefits present in other techniques, previously described. These benefits include that the process is almost completely automated, that the analytical output has a high information content about the binding events, including specificity and relative binding affinity, and that it is possible to study the native biomolecular binding interactions of conformational epitopes brought together only in the correctly folded protein form. SPR also offers the possibility of directly observing the antigen-antibody binding interactions without the need of labeling reactions, saving time and manual labor. On the other hand, the disadvantages of SPR include the possible alteration of the binding interactions as a result of the antigen undergoing conformational changes due to its immobilization, the degradation of the antigen in a regeneration step, a lower sensitivity compared to binding assays such as ELISA, and a high cost of equipment (and therefore often high cost per sample) [44,45,46]. Within the field of antibodies, most SPR applications use antibodies (or antigens) bound to a gold surface of a sensor chip. Binding interactions of added antigens (or antibodies) are monitored real-time by measuring the change of refractive index using polarized light [44]. In the more specific field of snake antivenom antibodies, SPR has mainly been used for studying binding kinetics and affinity [47,48,49,50,51], but could also be exploited for the study of antivenom cross-reactivity. Recently, a study by Langer et al. [52], investigating the cross-reactivity of polyclonal and monoclonal antibodies for synthetic cannabinoids, demonstrated the potential of SPR as a valuable tool for this type of investigation due to the reduced amount of manual labor, the real-time analysis, and the high information content obtained about the binding interactions. Also, a newly published study by Choudhury et al. [53] has revealed the development of a portable and inexpensive SPR measurement device with an integrated biosensor for detection of snake venom proteins, and illustrated the use of this device in the study of snake venom protein-antibody interactions. Such a device could hold great potential in evaluating antivenom/antibody cross-reactivity against snake toxins, especially due to its lowered cost.

## 4. Antivenomics

With the introduction of powerful mass spectrometry approaches in venom research, novel methodologies have emerged, which facilitate more holistic and high-throughput assessments of cross-reactivity of toxin-targeting antibodies [11,54], which may limit the use of rodent assays [55]. Antivenomics is a proteomics-based approach [56] designed to support in vivo and in vitro preclinical tests [57,58] in the qualitative and quantitative characterization of the immunological profile and the extent of cross-reactivity of antivenoms towards venoms [55]. However, for the application of antivenomics, a comprehensive characterization of the toxin profile of a venom of interest through proteomic, transcriptomic, and/or genomic studies (i.e., venomics) is required [59]. Therefore, the venom first needs to be fractionated using reversed-phase high performance liquid chromatography (RP-HPLC), during which the venom is forced through a column filled with solid adsorbent material. Each toxin will interact with this material slightly differently (according to its polarity) and therefore have a different flow rate through the column, resulting in the venom being separated into the different venom fractions. Upon further separation using gel electrophoresis, venom fractions can be analyzed using proteomics (involving mass spectrometry, MS), where the identity of each toxin present in the venom fractions can be determined. The same approach applies to antivenomics, with the difference that the venoms analyzed have previously been incubated with antivenoms.

**Figure 2:**
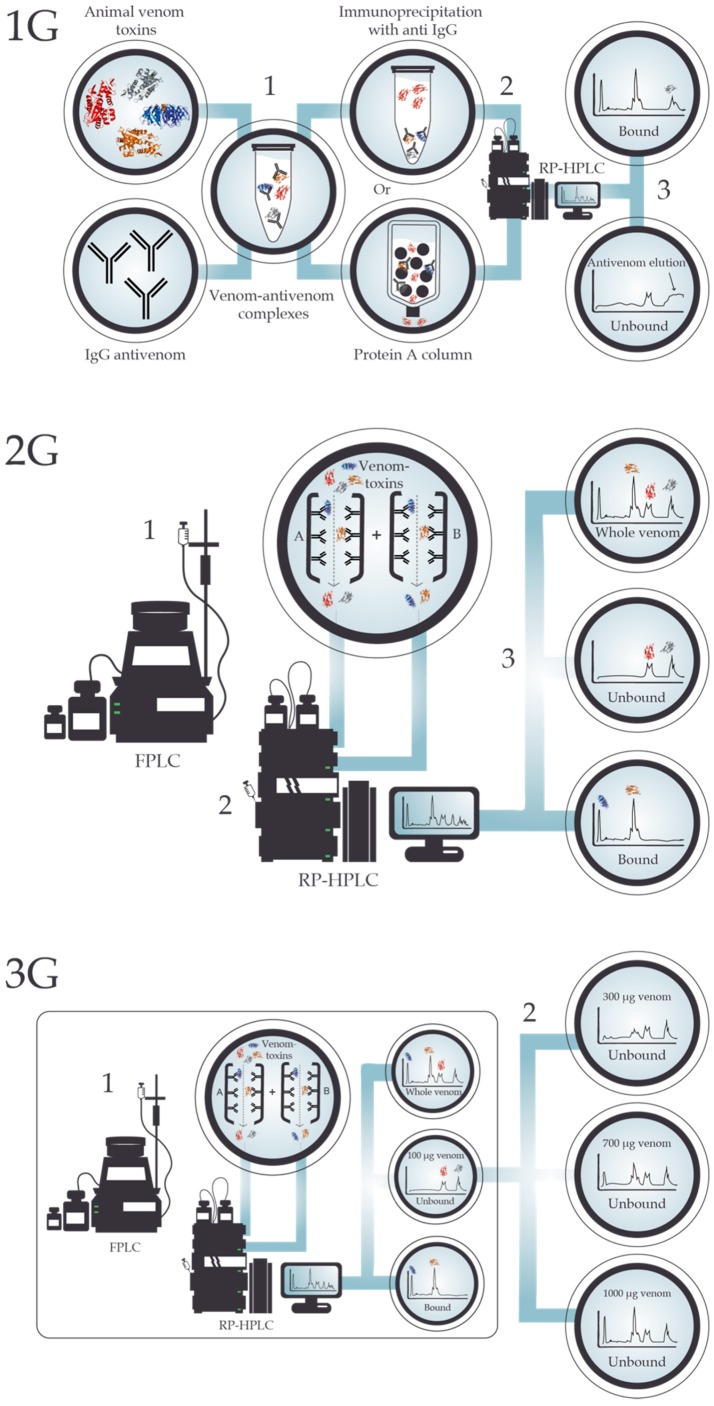
The evolution of the antivenomics methodology. (**1G**) First generation antivenomics involves the formation of toxin-antibody complexes (1), which are then either immunoprecipitated or forced through a Protein A column to separate toxin-antibody complexes from unbound toxins (2). Both fractions are then analyzed by reversed-phase high performance liquid chromatography (RP-HPLC), which allows for the identification of bound and unbound toxins. (**2G**) In second generation antivenomics, antivenom molecules (whole antibodies or fragments) are covalently immobilized onto a chromatographic matrix (immunoaffinity column), and the venom of interest is forced through this column, with the unbound toxins running straight through (1). Thereafter, the bound and unbound toxins are analyzed by RP-HPLC (2). Finally, quantitative comparisons of RP-HPLC chromatograms of whole venom and the immunoaffinity column eluates can be made, thus providing qualitative and quantitative information on both the set of toxins bearing antivenom-recognized epitopes and those toxins exhibiting poor immunoreactivity (3). (**3G**) Third generation antivenomics builds upon the concept of the immunoaffinity column applied in 2G antivenomics and repeats the same initial procedure (1). However, a range of venom-antivenom concentrations are tested to determine the maximal binding capacity of an antivenom against each toxin in a venom, as well as the quantification of the fraction of toxin-specific antibodies present in the antivenom (2).

In its original iteration, also known as “first generation antivenomics” (1G), the approach was based on in-solution immunoprecipitation of antigen-antibody complexes, followed by chromatographic quantification of the free antigen present in the supernatant, as seen in Figure 2, 1G. This allowed a time efficient protocol for in vitro investigation of cross-reactivity of several antivenoms against different venoms [56,60,61,62,63,64,65]. As an example, Lomonte et al. [56] demonstrated how the ICP polyvalent antivenom, PoliVal-ICP, was able to immunoprecipitate important venom toxins from both *Bothriechis lateralis* and *B. schlegelii*, despite a significant difference in the venom proteins between the two taxa and, more importantly, despite the venoms used for the production of the antivenom being from very different snake species (i.e., *Bothrops asper*, *Crotalus simus*, and *Lachesis stenophrys*). On the other hand, they also found that the antivenom demonstrated only limited recognition capability of certain venom components present in either or both *Bothriechis* species [56].

First-generation antivenomics is limited in its potential applications, since it is mainly suitable for whole IgG antivenoms and suboptimal for determining the immunoreactivity profile of an antivenom towards low abundance toxins [66]. A re-design of the methology was introduced to enable the assessment of F(ab’)_2_ and Fab-based antivenoms, which cannot be assessed through immunoprecipitation with anti-IgG or Protein A columns, and improve the detectability of low abundance toxins, coined “second generation antivenomics” (2G) [66]. 2G antivenomics involves the immobilization of the antivenom molecules on a chromatographic matrix to generate an immunoaffinity column, as seen in Figure 2, 2G [66]. This is achieved by filling a column with Sepharose, adding a coupling buffer, and then incubating the column for 4 hours with antivenom IgG molecules [66]. 2G antivenomics is accompanied by additional advantages, such as providing direct information on both the non-bound and bound toxins. This allows for a smoother baseline of the surrogate chromatograms of the affinity column fractions and reusability of the affinity columns, which contributes to the economy and reproducibility of the method [66]. Indeed, shortly after the implementation of 2G antivenomics in 2012 [66], studies were taking advantage of these technological advancements [67,68,69,70,71]. For instance, in 2012, Fahmi et al. [67] investigated the immunoreactivity profile of an experimental monospecific antivenom, CcMo_AV, and a commercial F(ab’)_2_ antivenom, Gamma-VIP, towards geographic variants of *Cerastes cerastes* and *C. vipera* venoms. They detected similar immunocapturing capabilities towards Moroccan, Tunisian, and Egyptian *C. cerastes* venom proteins. Furthermore, they were able to identify the proteins escaping immunorecognition as all being PLA_2_s, and that there was only a low degree of cross-reactivity for CcMo_AV and Gamma-VIP antivenoms towards *C. vipera* venom toxins [67]. Consequently, second-generation antivenomics allowed the authors to quantitatively assess the relative proportion of toxins that were not bound by the antivenom and allowed for the conclusion that a more complete therapeutic coverage could be achieved by including *C. vipera* venom in the immunization mixture used for antivenom production.

In 2017, a further updated protocol was developed for antivenomics, “third generation (3G) antivenomics”, as seen in Figure 2, 3G [72]. This methodology was designed to enable the determination of the maximal binding capacity of an antivenom against each toxin in a venom, as well as the quantification of the fraction of toxin-specific antibodies present in the antivenom. This permitted improved estimates of the quantities of antivenom required to neutralize a certain amount of venom [72]. These improvements were achieved through the application of size exclusion HPLC, which can be advantageous compared to other chromatographic methods, as it conserves the native structure and binding characteristics of venoms and antivenoms [72]. This advancement in the analytical capabilities of antivenomics allows for a more functional and direct in vitro comparison of different antivenoms raised against venoms of the same species [72]. As an example of how functional toxin-binding of an antivenom can be assessed using 3G antivenomics, Pla et al. [72] investigated the efficacy of the EchiTAb-Plus-ICP antivenom against toxins from *Bitis arietans* and revealed distinct concentration-dependent patterns of maximal binding. Further observations of importance were made when assessing the efficacy of the same antivenom against *Naja melanoleuca* venom, which demonstrated that many of the toxins were recognized, albeit at varying venom:antivenom ratios [72]. However, the highly toxic 3FTxs were not recognized, thus indicating that the use of EchiTAb-Plus-ICP against bites by *N. melanoleuca* would likely not have a positive medical impact on the victim, as 3FTxs are the key components of this venom in terms of toxicity [28]. This suggests, that by combining 3G antivenomics with toxicovenomic analyses of a venom, it may be possible to substitute a large number of the in vivo tests traditionally performed to assess the cross-neutralization potential of antivenoms [55].

Despite its relatively recent introduction, numerous studies aimed at assessing the immunological profiles of antivenoms from different manufacturers and a range of different snake species from Europe, Asia, Oceania, Latin America, and Africa have demonstrated the usefulness and validity of antivenomics in complementing existing assays [67,68,73,74,75,76,77,78,79,80,81,82]. However, antivenomics can still merely suggest likely explanations of observed cross-reactivity, such as the presence of the same subfamily of important toxins in two given venoms, where one venom is used in antivenom production [83]. Antivenomics cannot explain antivenom cross-reactivity at the epitope level [54].

## 5. High-Density Peptide Microarray

To improve the understanding of molecular interactions between snake venom toxin epitopes and antivenom antibody paratopes, immunoassay quantification of antivenom binding to immobilized synthetic peptides have in more recent years been used to identify which toxin sequences contain linear elements of epitopes recognized by given antivenoms. These peptide (micro)-arrays involve peptides designed to represent individual segments of the amino acid sequence of a given snake toxin [84,85,86,87,88]. They can therefore be used to potentially explain the origin of any observed venom cross-reactivity or even para-specificity from wet lab studies such as antivenomics. The most common method for synthesizing peptide microarrays has been SPOT synthesis, where peptides are synthesized using solid-phase peptide synthesis on the base of a cellolose membrane [46]. The SPOT method has been used to identify linear elements of a small number of snake toxins, but has also been applied to mapping the preferences and cross-reactivity patterns of antibodies targeting groups of selected toxins from *Tityus* scorpions [89,90,91] and *Loxosceles* spiders [92]. Although providing important molecular insight, studies using SPOT synthesis are limited to the investigation of a small selection of toxins and commercial antivenoms at a time, due to a low throughput.

Within the last couple of years, high-density peptide microarray technology (HDPMT) has been adapted to the study of snake toxin epitopes [18,24,25]. Compared to SPOT synthesis, the high-throughput epitope mapping approach made available with HDPMT allows for the analysis of a much higher number of different toxins and multiple antivenoms simultaneously at a relatively low cost per data point. HDPMT enables the identification of amino acid specific antibody-epitope interaction sites and may provide information on shared recognition sites between homologous toxins, and thereby to some extent explain observed cross-reactivity [18,24,46,93,94,95]. 

**Figure 3:**
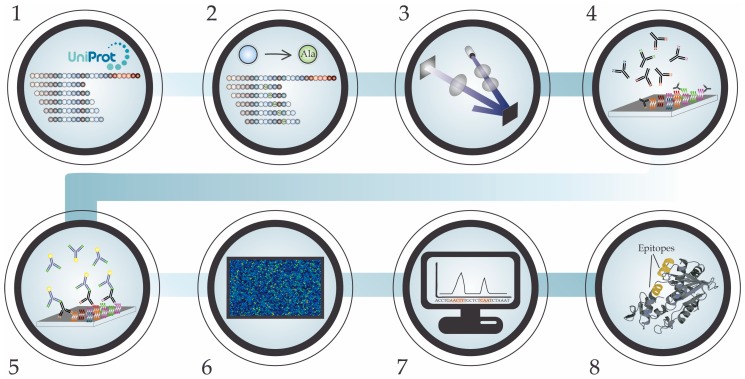
A stepwise visualization of the methodology behind the high-density peptide microarray approach. (**1**) In silico generation of a peptide library of *k*-mers, typically 7–16 amino acids in length, by tiling known sequences of snake venom toxins. (**2**) Expansion of the peptide library with point substitutions of every amino acid with a substitute amino acid, such as alanine. (**3**) Production of the microarray by mask-less photolithographic synthesis of peptides on a solid phase treated glass slide. (**4**) Binding of primary antivenom antibodies to peptides. (**5**) Binding of secondary fluorophore-labeled antibody to primary antivenom antibodies. (**6**) Image capture of the fluorescent signals and calculation of signal intensities. (**7**) Bioinformatic data normalization, analysis, and epitope mapping. (**8**) Visualization of the potential conformation of linear epitopic elements in generated 3D toxin models.

Each HDPM study begins with the generation of a peptide library. When studying the cross-reactivity of snake antivenoms, the library is designed in silico from a selection of known snake venom toxin amino acid sequences, originating from snake species of interest. A library of *k*-mers of typically 7–16 amino acid residues are generated based on the amino acid sequence of the proteins of interest and through a selected tiling interval, as seen in Figure 3, step 1 [18,24]. The resulting peptide library can then also be extended to include a scan of point amino acid substitutions, where each amino acid residue in each peptide is turn-wise substituted (e.g., to alanine), as seen in Figure 3, step 2 [18,24]. The methodology behind the peptide synthesis of HDPMT has gone through many evolutionary steps. However, each microarray is now usually prepared through mask-less photolithographic synthesis of peptides on a solid phase treated glass slide, as shown in Figure 3, step 3 [96]. In this type of synthesis, 365 nm light is projected onto amino groups on a glass slide, covered by photolabile protection groups, in patterns corresponding to the synthesis fields. The projected patterns are generated using digital micromirrors and projected onto the synthesis surface using UV-imaging optics. The relevant amino acid additions are coupled to projected predefined fields after UV-induced removal of the photoprotection groups in said fields [96,97]. After peptide synthesis, the microarrays are incubated with a snake antivenom of interest (or control), after which unbound antibodies are washed away, as shown in Figure 3, step 4. This is followed by a second incubation step with a fluorophore-labeled secondary antibody (a fluorophore-labeled anti-horse IgG in case of equine antivenoms), which binds to the primary antibodies from the snake antivenom. Once again, the unbound antibodies are washed away, as shown in Figure 3, step 5. Thereafter, an image of the fluorescent signal is recorded using a laser scanner, and the signal intensity is calculated from the resulting image, using specialized software, as shown in Figure 3, step 6 [18]. The produced data of the antivenom antibody binding signal intensities can then be bioinformatically normalized, analyzed, and mapped back to the known snake toxin sequences to provide insight into which toxin linear epitopic elements are recognized by the antivenom antibodies, as shown in Figure 3, step 7. Antivenom cross-reactivity is detected when the tested antivenom recognizes linear epitopic elements on toxins originating from more than one snake species. If the antivenom recognizes linear epitopic elements on toxins from venoms of snake species not used in the production of the antivenom, the results indicate the presence of antivenom para-specificity [18,24]. The detected linear epitopic elements can subsequently be visually displayed in generated 3D structures of the analyzed venom toxins to identify any potential conformational structures affecting the area, where the epitope element was found, as shown in Figure 3, step 8 [18,24,25].

Previous studies have already demonstrated the potential of HDPMT in the field of snake antivenom cross-reactivity research, such as Engmark et al. [18], which, for the first time, demonstrated how this technology could be used to identify linear epitopic elements in snake venom toxins. In that study, the researchers identified toxin epitopes recognized by antivenom antibodies for all, but two, manually curated toxins from every mamba species and four cobra species, endemic to sub-Saharan Africa. The study also unveiled how common epitopic elements could not be identified by the HDPMT for type 1 and type 2 α-neurotoxins, which both target the nicotinic acetylcholine receptor. Therefore, the study hypothesized that no, or very little, antibody cross-recognition takes place between the two types of α-neurotoxins, leading to the highlighted importance for an efficacious antivenom to recognize and neutralize the specific subtype of α-neurotoxins present in the venom of the snake species the antivenom is intended to work against. The study demonstrated the presence of cross-reactivity for the SAIMR antivenom, produced by South African Vaccine Producers Ltd., towards six different type 1 α-neurotoxins included in the study as well as para-specificity towards three additional type 1 α-neurotoxins from the venoms of *Dendroaspis viridis* and *Naja haje*. This para-specificity was thought to be derived from sequences in the *D. viridis* and *N. haje* toxins that were shared with the other six type 1 α-neurotoxins included in the immunization mixture used to produce the SAIMR antivenom. The study also showed how para-specificity can be lost towards a type 2 α-neurotoxin from *D. viridis* (α-elapitoxin-Dv2a, UniProt ID: P01395), when two amino acid substitutions (Asp64, Lys69) are introduced in the C-terminal, resulting in another type 2 α-neurotoxin from *D. viridis* (α-elapitoxin-Dv2b, UniProt ID: P01394). Thereby, the researchers illustrated how high similarity between toxins is insufficient for predicting antibody binding if the key residues for the interaction are unknown.

In another study by Engmark et al. from 2017 [24], cross-reactivity of the ICP polyvalent antivenom was investigated against enzymatic viper toxins. Here, it was demonstrated that the PoliVal-ICP bound to linear B-cell epitopes positioned outside of the active site, in locations related to tissue interaction or allosteric inhibition in Snake Venom Metalloproteinases (SVMPs), PLA_2_s, and Snake Venom Serine Proteinases (SVSPs). In addition, similar to their previous findings [18], it was discovered that substitution of key amino acid residues in the sequence of the identified linear epitopic elements could result in the loss of antivenom cross-recognition.

In a study using a slightly different approach, Engmark et al. [25] identified a single linear epitopic element in a short neurotoxin from black mamba venom (UniProt ID: P01416), using antiserum derived from a rabbit immunized with this single toxin. The study also found the rabbit antiserum to exhibit very limited cross-reactivity to the majority of 751 other peptides from 3FTxs and dendrotoxins tested in the study. They therefore concluded that the antibodies raised in the immunized rabbit expressed very high selectivity against the single short neurotoxin, in contrast to polyvalent antivenoms tested by Engmark et al. [18,24]. Thereby, this study indicated that antivenom cross-reactivity may likely not be a trait that is easily achieved, but instead can only be attained via rational and systematic immunization schemes using toxin mixtures (or through sophisticated antibody technologies).

In conclusion, these three studies have demonstrated the applicability of HDPMT for identification of both highly specific epitopes, where only a single toxin is recognized by an antivenom, and shared or conserved epitopes between homologous toxins. This may help guide the scientist by explaining when cross-reactivity, and to some extent para-specificity, is observed and when it is not. However, HDPMT does not provide information on antibody binding to more complex conformational epitope structures, as HDPMT is currently limited to the study of linear epitopic elements [98]. Conformational epitopes would in contrast be recognized, although not identified, in antivenomics studies of whole venom [83]. The possibility of combining antivenomics with a high-throughput epitope mapping approach of snake venom toxins, such as HDPM, to validate observed cross-reactivity and para-specificity may provide deeper insight into toxin-antivenom interactions. Such insight may further guide the development of broad-acting antivenoms with higher efficacy and polyvalence [54,99].

## 6. Outlook

The phenomenon of antivenom cross-reactivity has been studied for many decades with a range of conventional techniques, such as immunodiffusion, immunoblotting, ELISA, in vivo neutralization assays, and surface plasmon resonance. In recent time, however, the introduction of mass spectrometry, proteomics, and efficient peptide synthesis has enabled the development of new and powerful techniques that can be harnessed to study the ability of both polyclonal and monoclonal antibodies to cross-bind different toxin targets. Two of these new techniques include antivenomics and high-density peptide microarray technology [55,100]. Antivenomics allows for thorough in vitro assessment of an antivenom’s ability to bind snake venom toxins in an efficient manner. However, this technique does not provide much insight into the specific interactions between the paratopes and epitopes responsible for binding between antibodies and toxins [54]. High-density peptide microarray technology is a complementary technique, which allows for very high-throughput assessment of specific binding interactions between linear elements of toxin epitopes and antibodies, although the binding strength can only be assessed semi-quantitatively [100]. In combination, antivenomics and high-density peptide microarray technology may help create in-depth knowledge on the fundamental mechanisms involved in antivenom cross-reactivity and broaden our understanding of how toxin-neutralization is achieved [54,55]. In turn, this may help guide expansion of use for existing antivenoms and the development of future antivenoms, as well as possibly becoming a key driver for building testable hypotheses on how toxins and antibodies interact and how neutralization occurs for different toxin families [6,24]. Such knowledge is relevant for conventional antivenom development and manufacturing, but may become even more essential for the development of next generation antivenoms, which follow a more toxin-centric instead of a species-centric development approach [101]. Expanding our knowledge and toolbox in the field of antivenom cross-reactivity will hopefully support antibody research in the field of toxinology and in other fields.
